# Virulent Newcastle disease virus genotypes V.3, VII.2, and XIII.1.1 and their coinfections with infectious bronchitis viruses and other avian pathogens in backyard chickens in Tanzania

**DOI:** 10.3389/fvets.2023.1272402

**Published:** 2023-10-19

**Authors:** Henry M. Kariithi, Jeremy D. Volkening, Gaspar H. Chiwanga, Iryna V. Goraichuk, Tim L. Olivier, Peter L. M. Msoffe, David L. Suarez

**Affiliations:** ^1^Exotic and Emerging Avian Viral Diseases Research Unit, Southeast Poultry Research Laboratory, U.S. National Poultry Research Center, Agricultural Research Service, USDA, Athens, GA, United States; ^2^Biotechnology Research Institute, Kenya Agricultural and Livestock Research Organization, Nairobi, Kenya; ^3^BASE_2_BIO, Oshkosh, WI, United States; ^4^Tanzania Veterinary Laboratory Agency, Mtwara, Tanzania; ^5^National Scientific Center Institute of Experimental and Veterinary Medicine, Kharkiv, Ukraine; ^6^Department of Veterinary Medicine and Public Health, Sokoine University of Agriculture, Morogoro, Tanzania; ^7^National Ranching Company Ltd., Dodoma, Tanzania

**Keywords:** Avastrovirus, IBV GI-19, ICPI, Newcastle disease, NGS, rRT-PCR

## Abstract

Oropharyngeal (OP) and cloacal (CL) swabs from 2049 adult backyard chickens collected at 12 live bird markets, two each in Arusha, Dar es Salaam, Iringa, Mbeya, Morogoro and Tanga regions of Tanzania were screened for Newcastle disease virus (NDV) using reverse transcription real-time PCR (rRT-PCR). The virus was confirmed in 25.23% of the birds (*n* = 517; rRT-PCR *C*_T_ ≤ 30), with the highest positivity rates observed in birds from Dar es Salaam region with higher prevalence during the dry season (September–November 2018) compared to the rainy season (January and April–May 2019). Next-generation sequencing of OP/CL samples of 20 out of 32 birds that had high amounts of viral RNAs (*C*_T_ ≤ 25) resulted in the assembly of 18 complete and two partial genome sequences (15,192 bp and 15,045–15,190 bp in length, respectively) of NDV sub-genotypes V.3, VII.2 and XIII.1.1 (*n* = 1, 13 and 4 strains, respectively). Two birds had mixed NDV infections (V.3/VII.2 and VII.2/XIII.1.1), and nine were coinfected with viruses of families *Astroviridae*, *Coronaviridae*, *Orthomyxoviridae*, *Picornaviridae*, *Pneumoviridae*, and *Reoviridae*. Of the coinfecting viruses, complete genome sequences of two avastroviruses (a recombinant chicken astrovirus antigenic group-Aii and avian nephritis virus genogroup-5) and two infectious bronchitis viruses (a turkey coronavirus-like recombinant and a GI-19 virus) were determined. The fusion (F) protein F_1_/F_2_ cleavage sites of the Tanzanian NDVs have the consensus motifs ^112^ RRRKR↓F ^117^ (VII.2 strains) and ^112^ RRQKR↓F ^117^ (V.3 and XIII.1.1 strains) consistent with virulent virus; virulence was confirmed by intracerebral pathogenicity index scores of 1.66–1.88 in 1-day-old chicks using nine of the 20 isolates. Phylogenetically, the complete F-gene and full genome sequences regionally cluster the Tanzanian NDVs with, but distinctly from, other strains previously reported in eastern and southern African countries. These data contribute to the understanding of NDV epidemiology in Tanzania and the region.

## 1. Introduction

Virulent (neurotropic or viscerotropic velogenic) strains of Newcastle disease virus (vNDV, or avian paramyxovirus type 1; genus *Orthoavulavirus* of *Paramyxoviridae* family (1)) cause the highly contagious and fatal Newcastle disease of domestic poultry (ND; clinically manifested by hemorrhagic gastroenteritis, pneumonia, and/or encephalitis depending on factors such as virus strain, type/breed/age/immune status of the affected avian species, and other underlying external factors) with substantial economic burden in the poultry industry (2–5). Infections with vNDVs, defined as viruses with an intracerebral pathogenicity index (ICPI) score of at least 0.7 in 1-day-old chicks (*Gallus gallus*), or presence of multibasic amino acid residues and a phenylalanine residue at position 117 of the fusion protein cleavage site (^112^ [R/K]-R-Q-[R/K]-R↓F ^117^; ↓ indicate cleavage site) are reportable to the World Organization of Animal Health [WOAH or often referred to as Office International des Epizooties (OIE)] (6). Coinfections of NDVs with other viral pathogens (e.g., avian influenza virus, avian bronchitis virus, avian metapneumovirus) have been reported in poultry and other avian species (7–11), but their impacts on viral pathogenesis and ND remain unclear.

The polyhexameric negative-stranded RNA genome of NDV (length of either 15,186, 15,192, or 15,198 bases) encodes eight proteins: nucleocapsid protein (NP), phosphoprotein (P), matrix (M), fusion (F), hemagglutinin-neuraminidase (HN), and large polymerase (L; tandemly organized as 3′-NP-P-M-F-HN-L-5′ (12–14)), and two additional multifunctional accessory proteins (V and W) produced via co-transcriptional editing of the P-gene mRNA during active virus replication (15–17). The HN and F are the most important NDV glycoproteins because they mediate vital aspects of viral infection such as host-range, attachment to susceptible host cell, fusion of viral envelope to cell membranes, tissue tropism, and systemic viral spread within infected host (18–23). The NP, P and L proteins form the active viral RNA-dependent RNA polymerase (RdRp) complex, which is essential for genome synthesis (18, 24), while the M protein is involved in virion assembly and egress (18, 25).

Since the first report of highly pathogenic ND in the UK and Indonesia in the late1920s, panzootics have occurred in poultry (26), and the disease has extensively spread in the Middle East, Europe, the Americas and Africa (27). Newcastle disease is considered endemic and a major constraint to traditional poultry production in Africa; although few genomic datasets are available on the repertoire of the NDV strains in circulation on the continent, genotypes V and VII are frequently reported (28–33). With the rampant and severe ND outbreaks in many African countries, the continent is considered as a reservoir of novel vNDVs (34–36). This is significant because the socioeconomics of most rural African households is heavily dependent on small-scale backyard poultry composed predominantly of unvaccinated indigenous domestic chickens (*Gallus gallus domesticus*; ~ 80% of poultry flocks), which are traded at live bird markets (LBMs) to supplement incomes (37, 38). Further, rural African farmers are often unable to implement preemptive disease control measures because of the unpredictability of ND outbreaks, coupled with the rearing of heterogenous poultry populations that interact with wild birds, which are potential asymptomatic carriers of NDV (39–42).

In the present study, we report the detection and molecular characterization of vNDVs identified using nontargeted next-generation sequencing (NGS) of clinical chicken samples collected between September 2018 and May 2019 from LBMs in central, eastern (coastal), southern and northern regions in Tanzania, and virus isolation and characterization of a small subset of the samples. We also present genome sequence analyses of other viral agents that coinfected with the vNDVs in some of the sampled chickens.

## 2. Materials and methods

### 2.1. Samples

The clinical samples used in this study were collected from adult backyard chickens during a surveillance of NDVs conducted at 12 LBMs located in six administrative regions of Tanzania: Arusha in the north (central and Kilombero), Dar es Salaam on the east coast (Buguruni and Kisutu), Iringa in central (Mashine tatu and Miomboni), Mbeya in the south-west (Sokomatola and Soweto), Morogoro in the mid-east (Manzese and Mawenzi) and Tanga in the north-east (Ngamiani and Uzunguni). The LBMs in these regions are well-known for poultry trade in the country. There were two sampling periods conducted: September to November 2018 (dry season) and January and April–May 2019 (rainy season). From each chicken, an oropharyngeal (OP) and a cloacal (CL) swab was collected using sterile, plastic-shafted flocked swabs (Puritan Medical, Guilford, ME), each of which was placed in individual 2.0 ml Corning® cryogenic vials (Corning Inc., Corning, USA) containing 1.5 ml of viral transport media (brain-heart-infusion broth; Difco, NZ) according to standard procedures (43). Swabs were immediately stored in liquid nitrogen and preserved at −80°C until shipment to Southeast Poultry Research Laboratory (SEPRL) of the USDA-ARS in Athens, GA, USA for analyses. At the time of sampling, vaccination status or histories of the sampled birds were not available, and any observed clinical signs consistent with avian diseases were recorded.

### 2.2. RNA extraction and virus detection

Total RNAs were extracted individually from 50 μl of each OP and CL sample using the MagMAX™-96 AI/ND Viral RNA Isolation Kit (Thermo Fisher Scientific, MA, USA) on an automated KingFisher Magnetic Particle Processor (Thermo Fisher, USA) and eluted in 50 μl of elution buffer according to manufacturer’s instructions. The RNA extracts were used to detect NDV using real-time reverse transcription polymerase chain reaction (rRT-PCR) assays with primers and probes targeting the large polymerase gene (L-12200 test; detects all class II NDV strains) as previously described (44). Samples with rRT-PCR cycle threshold (*C*_T_) of ≤40 were designated as “suspect NDV-positive,” *C*_T_ ≤ 30 as “confirmed NDV-positive,” and *C*_T_ ≤ 25 as “strongly NDV-positive” (i.e., contained high amounts of viral RNAs). For further analysis using NGS (described below), a subset of OP samples with *C*_T_ ≤ 25 and their counterpart CL samples (i.e., from the same birds) were randomly selected and tested using rRT-PCR targeting a conserved region of the matrix gene (M-4100 test; detects diverse NDV strains), and the fusion gene (F-test; specifically detects potentially virulent NDV strains) as previously described (45, 46). All three rRT-PCR tests were performed using AgPath-ID one-step RT-PCR Kit (Ambion) and the ABI 7500 Fast Real-Time PCR system (Applied Biosystems, Waltham, MA) according to the manufacturer’s instructions.

### 2.3. Analysis of NDV positivity rates

Based on the rRT-PCR L-test, the differences in the NDV positivity rates across the sampling locations were determined using the Kruskal-Wallis test, and where significant differences (*p*-values of less than 0.05) were observed, Dunn’s and Tukey’s honest significant differences (TurkeyHSD) tests with Bonferroni correlations were performed. The correlation and statistical analyses were performed using analysis of variance (ANOVA) executed in RStudio version 2023.06.0.^1^

### 2.4. Assessment of NDV pathogenicity

A subset of the “strongly NDV-positive” OP samples (with *C*_T_ ≤ 25) was selected for assessment of pathogenicity using the standard ICPI test (47). Briefly, the viruses were isolated in 9-to-11-day-old specific pathogen free (SPF) embryonated chicken eggs (three eggs per sample; up to two blind passages), followed by harvesting of allantoic fluid (AF) at the end of a 7-days incubation period and hemagglutination assay (HA) testing. Then, 50 μl of diluted HA-positive AF samples (10-fold in PBS) were inoculated intracerebrally into 1-day-old SPF chicks (*n* = 10 birds per AF sample and sterile PBS-inoculated controls). The experimental chickens were monitored daily over an 8-day period for determination of the ICPI: strains with ICPI scores greater than 1.5 and between 0.7 and 1.5 were considered velogenic and mesogenic strains, respectively. All animal experiments were approved by local IACUC. Total RNAs from the AF samples were extracted and used for virus detection using the above-described rRT-PCR tests and subsequent NGS.

### 2.5. Next-generation sequencing

Our recently described in-house RNaseH rRNA depletion protocol (48) was used to selectively reduce the abundance of host-specific rRNAs (18S, 28S and mitochondrial) and bacterial rRNAs (16S/23S) in 12 μl of the total RNA samples selected as described above (OP, CL and AF samples). Sequence-independent, single-primer amplification (SISPA) (49) was used to prepare cDNAs from 10 μl of the RNaseH-treated RNAs using random K-8 N primer with the SuperScript ™ IV and Klenow polymerase (NEB Inc., USA) kits according to manufacturer recommendations. The Phusion® High-Fidelity PCR Kit (NEB Inc., USA) was used to amplify 5 μl of bead-purified (Agencourt AMPure XP Kit; Beckman Coulter Life Sciences, USA) cDNAs, followed by preparation of sequencing libraries using the Nextera ™ DNA Flex kit (Illumina, USA). After quantification using the Qubit™ dsDNA HS Assay (Thermo-Fisher Scientific) and Agilent 4,150 TapeStation HS D5000 (Agilent Technologies, Inc.) kits, the libraries were pooled (4 nM, 8 μl each), spiked with a control library (5% PhiX library v3), diluted to 10 pM final concentration and sequenced (paired-end; 2 × 300 bp) using the 600-cycle MiSeq Reagent Kit v3 (Illumina, USA) on an Illumina MiSeq instrument.

### 2.6. Sequence assembly

Raw NGS data were processed using a nontargeted classification and *de novo* assembly pipeline developed by BASE₂BIO LLC (Oshkosh, WI, USA). Specifically, for the consensus genome sequences published here, raw reads were pre-processed using Trim Galore v0.6.7^2^ to remove residual sequencing adapters, SISPA primers, and low quality 3′ ends (q < 8). The host (*Gallus gallus*) reads were removed using a BBTools^3^ bbduk filter against chicken genome build bGalGal1.mat.broiler.GRCg7b, with *k* = 35, hdist = 0, mincovfraction = 0.3. Final assembly was performed with MEGAHIT v1.2.9 (50) with default parameters. Assemblies were quality-reviewed by mapping trimmed reads against the assembly using BWA-MEM (51) with default parameters and inspecting with Integrative Genomics Viewer (52) for obvious coverage or assembly artifacts. Taxonomic classification was performed using KrakenUniq (53) with an in-house database composed of the host genome, potential vector contaminants, all viral sequences from GenBank, and a representative selection (minimum one per species) of full-length bacterial, archaeal, fungal, and protozoan genomes from NCBI. Read classifications were filtered using a patched version of the “krakenuniq-filter” package script, requiring a minimum taxon-specific *k-mer* fraction of 0.05 for viral taxa and 0.25 for all other taxa. Individual taxonomic identifications were further verified using BLASTn (54) search of *k-mer* classified reads against the `nt` database and subsequent lowest common ancestor (LCA) assignment using an in-house software.

### 2.7. Sanger sequencing

Internal gaps in the assembled consensus sequences (i.e., regions without read coverage) were filled using the SuperScript IV One-Step RT-PCR kit (Thermo Fisher Scientific, MA, USA) as recently described (55). The missing bases at the 3′- and 5′-termini were determined utilizing a single 3′-nt polynucleotide tailing reaction of both the genomic RNA and the full-length positive-sense antigenomic RNA. Briefly, the tailed RNA was used for a single reverse transcription reaction targeted to the common polynucleotide tails, followed by two separate PCRs each using one virus-specific primer and one targeted to a 19MER tag at the polynucleotide tail, as described previously (56). Sanger sequencing was performed using the BigDye™ Terminator v 1 Cycle Sequencing Kit on a 3,730 xl DNA Analyzer (Thermo Fisher Scientific, MA, USA) following the manufacturer’s instructions.

### 2.8. Sequence and phylogenetic analyses

The assembled consensus sequences were annotated using Geneious Prime® v2023.2.0 Beta^4^ as recently described (10, 11). Fusion glycoprotein cleavage site motifs were analyzed based on the WOAH code (6) and the presence of amino acid substitutions in the antigenic sites of the attachment (HN) glycoproteins were assessed (57). Sequences from this study were aligned with published NDV sequences (retrieved from GenBank using BLASTn algorithm) using MAFFT v7.490 (58), trimmed using trimAl v1.3 (59) and used for phylogenetic analysis using ML method in MEGA v 11.0.10 with the best model suggested by the program, 1,000 bootstrap replicates, and deletion of all positions with less than 95% site coverage at any position (60).

## 3. Results

### 3.1. Virus detection using rRT-PCR

**Figure 1:**
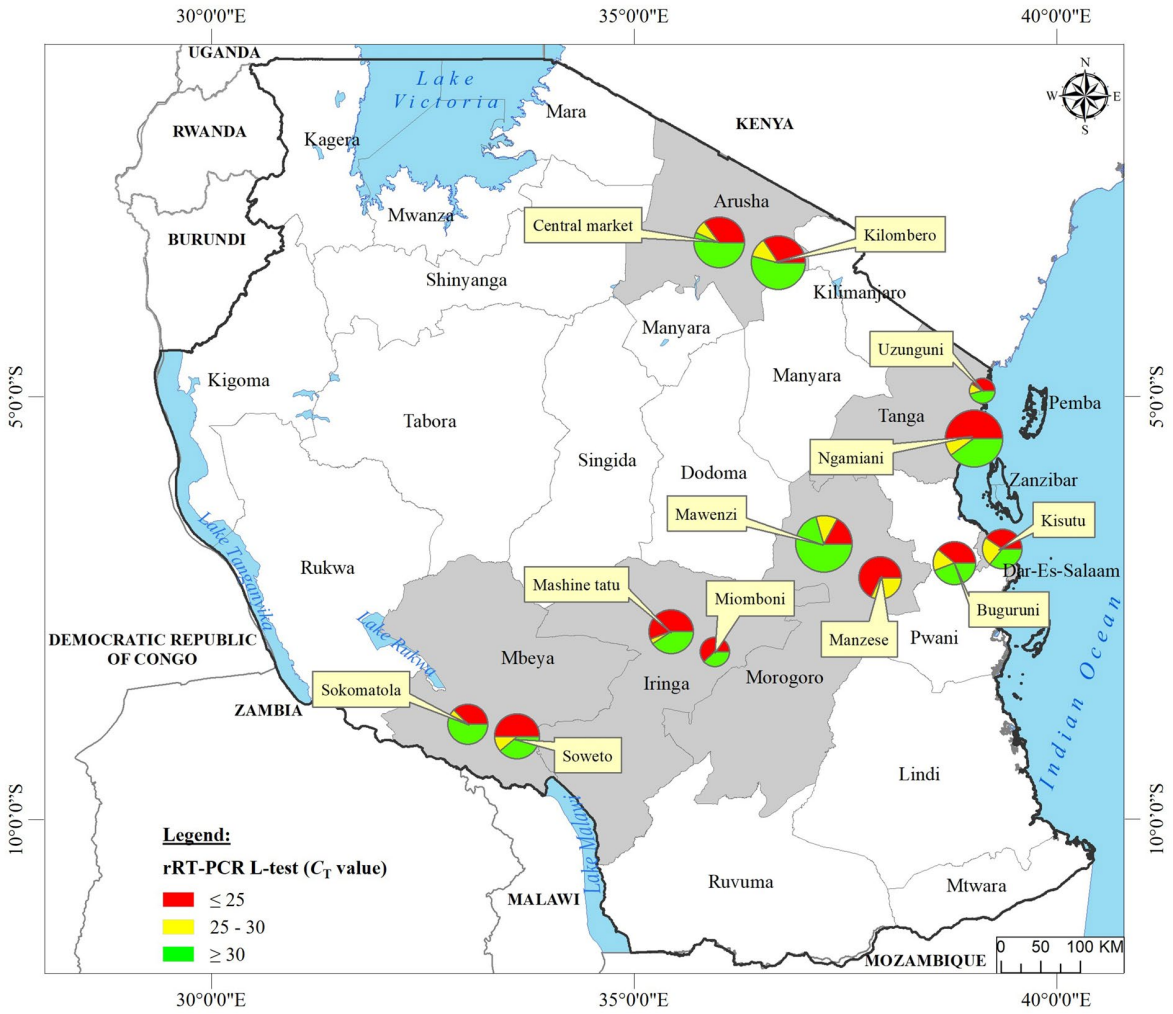
Locations of the 12 LBMs from where the backyard chicken samples analyzed in the current study were swabbed in six administrative regions (shown in gray background) of Tanzania. Oropharyngeal swabs from 2049 birds were screened for NDV using the rRT-PCR L-tests. For each sampling site, the numbers of chickens that tested NDV-positive are mapped using pie-charts to illustrate the rRT-PCR *C*_T_ values as described in the text, with the circle sizes corresponding to numbers of virus-positive samples as shown in the legend. Absolute numbers of NDV-positive samples are presented in Table 1.

**Figure 2:**
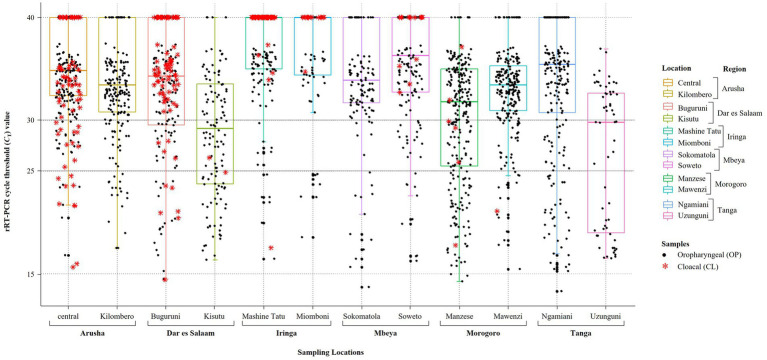
Plot of the results obtained from screening of backyard chickens for NDV infections using the rRT-PCR L-test. Oropharyngeal (OP) and cloacal (CL) samples from 2049 chickens were screened using the L-test, including all OP samples (*n* = 2049) and 407 CL samples, which are plotted using black full circles and red asterisks, respectively. The 12 LBM sampling locations in six regions of Tanzania are indicated on the x-axis and the rRT-PCR cycle threshold (*C*_T_) values are shown on the y-axis. For the plotting, samples that were NDV-negative by the rRT-PCR L-test were assigned a *C*_T_ cutoff value of 40 (shown on the *C*_T_ = 40 line on top of the plot). The averages of the *C*_T_ values for the OP samples are indicated by the boxplots, which are color-coded for each of the 12 LBMs. Samples with *C*_T_ below 25 were considered to be “strongly NDV-positive,” some of which were selected for further analysis using NGS as described in the text.

We analyzed OP and CL samples from 2049 birds; all the OP samples were screened using the rRT-PCR L-test resulting in the detection of NDV in 73.01% (*n* = 1,496; *C*_T_ cutoff of <40) of the samples across the 12 LBMs in six regions of Tanzania as shown in Figure 1. The NDV positivity rates were statistically significant between both the LBMs and the regions (*p*-value < 2.2^−16^; Figure 2). About 25% of these samples (*n* = 517) were confirmed as NDV-positive (*C*_T_ cutoff of ≤30), with the highest and lowest positivity rates observed in birds from Dar es Salaam and Iringa (42.91 and 8.09%, respectively) regions (Table 1). Considering specific LBMs, the positivity rates were generally higher during the dry than the rainy season, with the exception of Arusha region in the north where the positivity was higher during the rainy compared to the dry season, and in the Mbeya region in the south-west where samples were collected only during the rainy season. Of the confirmed NDV-positive samples, 51.84% (*n* = 268 out of 517) had high amounts of NDV RNAs (based on *C*_T_ cutoff of ≤25).

**Table 1:** Summary of the results obtained from screening for NDV infections in oropharyngeal (OP) samples from a total of 2049 backyard chickens analyzed in the current study.

Region	Location of LBM	Number of birds tested			Birds confirmed to be NDV-positive (*C*_T_ < 30)			Birds with high NDV RNA amounts (*C*_T_ < 25)^*^		
		Total	Dry season	Rainy season	Total	Dry season	Rainy season	Total	Dry season	Rainy season
Arusha	Central	421	100	100	72 (17.10%)	8 (8%)	18 (18%)	26 (36.11%)	0	6 (33.33%)
	Kilombero		105	116		19 (18.1%)	27 (23.28%)		5 (26.32%)	15 (55.56%)
Dar es Salaam	Buguruni	275	27	120	118 (42.91%)	15 (55.56%)	30 (25%)	61 (51.69%)	7 (46.67%)	11 (36.67%)
	Kisutu		12	116		9 (75%)	64 (55.17%)		6 (66.67%)	37 (57.81%)
Iringa	Mashine tatu	235	0	157	19 (8.09%)	0	12 (7.64%)	13 (68.42%)	0	6 (50%)
	Miomboni		33	45		6 (18.18%)	1 (2.22%)		6 (100%)	1 (100%)
Mbeya	Sokomatola	292	0	132	49 (16.78%)	0	21 (15.91%)	22 (44.90%)	0	12 (57.14%)
	Soweto		0	160		0	28 (17.5%)		0	10 (35.71%)
Morogoro	Manzese	522	121	161	167 (31.99%)	49 (40.5%)	68 (42.24%)	84 (50.29%)	23 (46.94%)	46 (67.65%)
	Mawenzi		116	124		25 (21.55%)	25 (20.16%)		4 (16%)	11 (44%)
Tanga	Ngamiani	304	100	145	92 (30.26%)	39 (39%)	21 (14.48%)	62 (67.39%)	24 (61.54%)	14 (66.67%)
	Uzunguni		0	59		0	32 (54.24%)		0	24 (75%)
Total		2,049	614	1,435	517 (25.23%)			268 (51.84%)		

The samples were collected from 12 live bird markets (LMBs) in six regions in Tanzania. Sampling was performed during the dry season (September–November 2018) and rainy season (January and April–May 2019). Samples with rRT-PCR cycle threshold (C_T_) values of ≤30 and ≤ 25 were considered to be “confirmed” and “strongly” NDV-positive, respectively, as described in the text.

* These are numbers (and %) of birds out of the “confirmed” cases, i.e., with *CT* values of ≤ 30.

Only 406 CL samples were rRT-PCR L-tested, and majority of those found to be NDV-positive (68.18%, *n* = 90 out of 132) had high *C*_T_ values (> 30). Only 276 of the 2049 birds had both their OP and CL samples rRT-PCR-screened, out of which 38.41% (*n* = 106) were NDV-positive on both sample types, but the amounts of NDV RNAs were higher in the OP compared to the CL samples, with positivity rates of 30.19 and 17.14% (*n* = 32 and 18 out of the 106 chickens), respectively, based on the *C*_T_ cutoff of ≤25 (see Supplementary Table S1).

### 3.2. Nontargeted virus discovery

NGS was performed on both OP and CL samples from 20 out of the above-mentioned 32 birds with high amounts of viral RNAs; NDV-specific RNAs were detected by NGS in all 20 OP samples and in 11 of the CL samples (Supplementary Table S2). The nine CL samples without detectable NDV RNAs were noted to have had either high *C*_T_ values (>30; *n* = 6) or were rRT-PCR-negative (*n* = 2), or unavailable (*n* = 1). Nine out of the 20 NDV-positive birds were coinfected with other viral agents belonging to the families *Astroviridae* [chicken astrovirus (CAstV) and avian nephritis virus (ANV)], *Coronaviridae* [infectious bronchitis virus (IBV)], *Picornaviridae* [chicken megrivirus (ChMeV) and sicinivirus type A (SiV-A)], *Pneumoviridae* [avian metapneumovirus type B (aMPV-B) and *Reoviridae* (avian rotavirus type G (AvRV-G)]. In addition to the viral agents, bacterial species of avian interest from six genera (*Avibacterium*, *Campylobacter*, *Enterococcus*, *Gallibacterium*, *Mycoplasma*, and *Ornithobacterium*) were identified, with the OP samples from all 20 birds containing species from multiple genera (Supplementary Table S2). The most common taxa present were *Avibacterium* spp. (*n* = 18 birds) and *Enterococcus* and *Campylobacter* spp. (*n* = 14 birds), while *Ornithobacterium rhinotracheale* (ORT) and *Mycoplasma synoviae*/*gallisepticum* were only present in the OP samples (*n* = 11 birds).

### 3.3. Genome sequences of Tanzanian NDVs

#### 3.3.1. Assembly

As summarized in Table 2, the higher numbers of NDV-specific NGS reads obtained from 18 out of the 20 birds (ranging from about 16,000 to 471,00 reads) allowed for the *de novo* assembly of 18 full-length and two partial genome sequences with sufficient read depths (~550-9714× range) and completeness (100% in 10 sequences of 15,192 bp in length; 99.03–99.99% in 10 sequences of 15,045–15,190 bp in length). Because of insufficient read depth (less than 2X) at various genomic positions, the consensus sequences assembled from two of the 20 birds are not further analyzed in this paper. The 10 partial genome sequences had either internal gaps and/or missing bases at their 3′-/5′-termini due to either ambiguous nucleotides, absence of reads or insufficient coverage. Sanger sequencing successfully filled the 35-nt and 11-nt internal gaps in the two 2,110-K103 sequences, the 6-nt gap in sequence 2,144-BD117, the 30-nt internal gap (but not the 6-nt gap) in sequence 1998-B08, 101 nt of the 195-nt-long gap in sequence 2001-B20, and the missing bases at the 5′-/3′-termini of the two 2,110-K103 sequences (Table 2).

**Table 2:** Summary of NDV genome assembly described in the current study.

Strain	Sample collection date	Region (location)	rRT-PCR L-test (*C*_T_)	NDV-specific reads	Median cov. depth	Consensus seq. length	% Completeness; gaps (# of missing bases at 5′-end|internal|3′-end)	Sub-genotype	Pathotyping		GenBank accession number
									ICPI^b^	F protein cleavage motif	
2014-E734	10-Oct-18	Morogoro (Manzese)	16.54	84,480	1,886	15,045	99.03%; (117|0|30)	VII.2	N/A	RRRKR↓FI	OR230617
2,110-K103	22-Sep-18	Dar es Salaam (Kisutu)	16.37	86,610	837	15,170	99.86%; (22|35|0)	V.3	N/A	RRQKR↓FV	OR230621
					1,056	15,169	99.85%; (0|11|23)	VII.2	N/A	RRRKR↓FI	OR230622
2,111-K104	22-Sep-18	Dar es Salaam (Kisutu)	16.37	375,478	9,828	15,192	100%	XIII.1.1	N/A	RRRKR↓FI	OR230623
2,115-M581	5-Oct-18	Morogoro (Mawenzi)	17.80	143,124	3,926	15,190	99.99%; (0|0|2)	VII.2	N/A	RRRKR↓FI	OR230624
2,144-BD117	24-May-19	Dar es Salaam (Buguruni)	16.82	32,728	631	15,187	99.97%; (0|6|5)	VII.2	N/A	RRRKR↓FI	OR230625
2,145-CA127	3-May-19	Arusha (Central)	16.79	177,140	3,947	15,178	99.91%; (13|0|1)	VII.2	N/A	RRRKR↓FI	OR230626
2,152-IM184	11-Apr-19	Iringa (Mashine tatu)	16.45	107,852	2,205	15,159	99.78%; (4|0|29)	VII.2	N/A	RRRKR↓FI	OR230627
2,159-ME50	29-May-19	Morogoro (Manzese)	14.25	63,407	1,206	15,177	99.90%; (10|0|5)	VII.2	N/A	RRRKR↓FI	OR230628
1995-B01^a^	21-Sep-18	Dar es Salaam (Buguruni)	18.55	73,140	1,389	15,192	100%	VII.2	N/A	RRRKR↓FI	N/A
				230,172	5,778	15,192	100%	XIII.1.1	N/A	RRRKR↓FI	N/A
1996-B03	21-Sep-18	Dar es Salaam (Buguruni)	15.80	390,295	8,984	15,192	100%	VII.2	1.70	RRQKR↓FV	OR230611
1997-B06	21-Sep-18	Dar es Salaam (Buguruni)	20.45	168,371	3,768	15,192	100%	VII.2	1.71	RRQKR↓FV	OR230612
1998-B08AF	21-Sep-18	Dar es Salaam (Buguruni)	14.92	471,551	9,928	15,156	99.76%; (0|30 + 6|30)	XIII.1.1	1.71	RRRKR↓FI	OR230613
2000-B18	21-Sep-18	Dar es Salaam (Buguruni)	14.89	392,000	8,910	15,192	100%	XIII.1.1	1.76	RRRKR↓FI	OR230614
2001-B20	21-Sep-18	Dar es Salaam (Buguruni)	17.13	16,849	383	15,084	99.29%; (108|95|0)	XIII.1.1	1.76	RRRKR↓FI	OR230615
2007-E713	10-Oct-18	Morogoro (Manzese)	13.98	213,901	5,081	15,192	100%	VII.2	1.66	RRRKR↓FI	OR230616
2015-E735	10-Oct-18	Morogoro (Manzese)	15.71	425,238	10,764	15,192	100%	VII.2	1.75	RRRKR↓FI	OR230618
2016-E736	10-Oct-18	Morogoro (Manzese)	16.62	350,402	7,934	15,192	100%	VII.2	1.88	RRRKR↓FI	OR230619
2017-E740	10-Oct-18	Morogoro (Manzese)	14.79	214,867	4,318	15,192	100%	VII.2	1.77	RRRKR↓FI	OR230620

Virus intracerebral pathogenicity index (ICPI) pathotyping determined using oropharyngeal (OP) swabs from nine of the 20 chickens are shown.

a The assembled genome sequences of the sub-genotypes VII.2 and XIII.1.1 strains identified in the chicken ID 1995-B01AF were not submitted to GenBank due to heterogeneity in various genomic positions (see text for details).

b N/A means that the ICPI was not determined for these samples.

Similar to other NDVs, the genomes of the Tanzanian strains identified in the current study consist of six open reading frames (ORFs) encoding six different proteins in the order of 3′-NP-P-M-F-HN-L-5′; the lengths of the CDS and deduced proteins are also consistent with other NDVs: N (1,470 nt; 489 aa), P (1,188 nt; 395 aa), M (1,095 nt; 364 aa), F (1,662 nt; 553 aa), HN (1716 nt; 574 aa), and L (6,615 nt; 2,204 aa).

#### 3.3.2. Classification and pathotyping

Sequence annotations and analyses showed that the Tanzanian NDVs identified in the current study are of sub-genotypes V.3, VII.2, and XIII.1.1 (*n* = 1, 13 and 4 strains, respectively); two birds from Kisutu and Buguruni LBMs in the Dar es Salaam region (chicken IDs 2,110-K103 and 1995-B01) had mixed infections with sub-genotypes V.3/VII.2 and VII.2/XIII.1.1, respectively (Table 2). Sub-genotypes VII.2 and XIII.1.1 sequences from sample 1995-B01 were highly heterogeneous in several genomic positions; because these could not be confidently resolved, they are not discussed further in this paper. The F protein sequences of the Tanzanian sub-genotype VII.2 strains have five basic aa residues in the F_1_/F_2_ cleavage site (^112^ R-R-R-K-R↓F ^117^) compared to four residues in the sub-genotypes V.3/XIII.1.1 strains (^112^ R-R-Q-K-R↓F ^117^); both motifs are consistent with vNDVs (61);. The virus was isolated in eggs from a subset of nine of the 20 OP samples and the virulence confirmed by ICPI scores ranging from 1.66 to 1.88 (Table 2).

#### 3.3.3. Genomic features of the protein-coding regions

**Figure 3:**
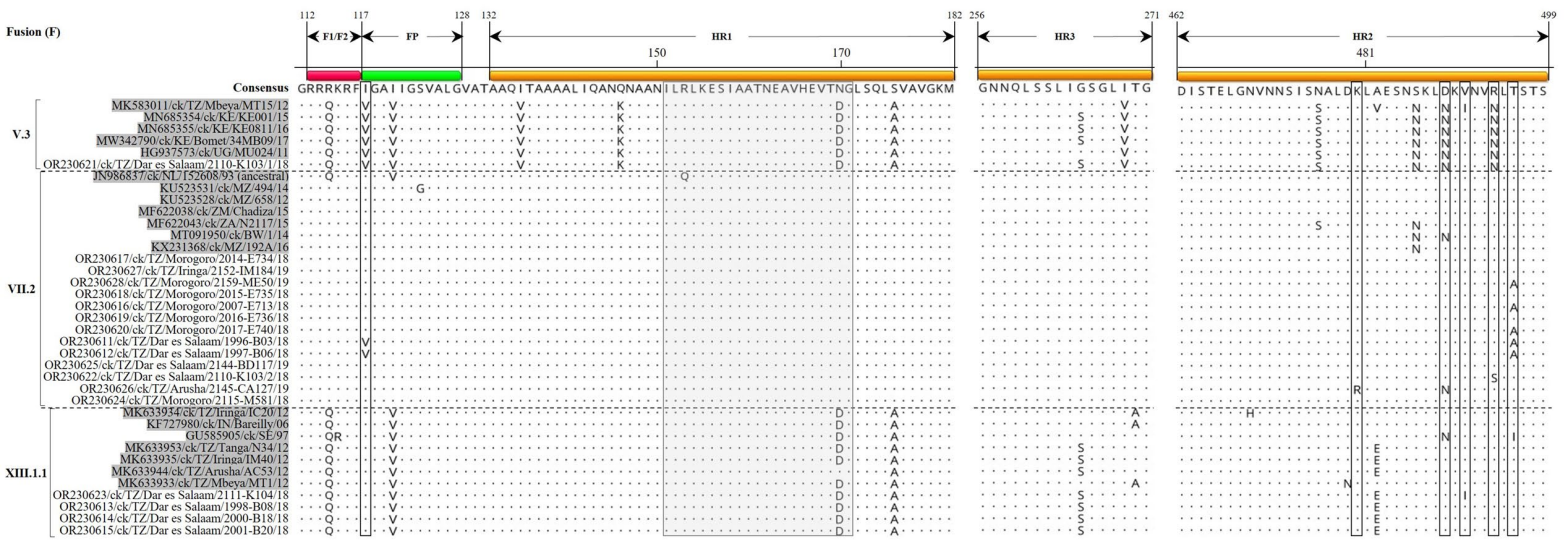
Alignment of the fusion protein amino acid (aa) residues in the F_1_/F_2_ cleavage site, fusion peptide (FP) and the heptad repeat regions 1–2 (HR1/2) of the Tanzanian strains identified in the current study and a selection of other V.3, VII.2, and XIII.1.1 strains (shaded in gray color). Residues in the consensus sequence are numbered relative to the first methionine residue (M1) of the translated protein sequences. The vertical dotted lines separate the sub-genotypes. The gray box (residues 151–171) and the open boxes indicate antibody neutralizing epitope region variations in the aa residues when comparing Tanzanian and other strains of the same sub-genotypes. Dots indicate identical aa residues.

The Tanzanian sub-genotype V.3F protein sequences from the current study have five *N*-linked glycosylation sites (N85, N191, N471, N494, and N541) compared to four sites in VII.2 (N85, N191, N471, and N541) and three in XIII.1.1 (N85, N191, and N471) strains. All Tanzanian V.3 and VII.2F protein sequences reported here have 12 conserved C residues (C27, C76, C199, C338, C347, C362, C370, C394, C399, C401, C424, and C523), but the four XIII.1.1 strains have C27R substitution. Except for a few substitutions, the fusion peptide (FP) and heptad repeat (HR) regions of the F protein sequences are largely conserved in strains of the same sub-genotypes (Figure 3).

High conservation was noted in the regions of NP protein sequences: region 1 (^171^ QVWVTVAKAMT ^181^; except for V172I and V174I substitutions in VII.2 and XIII.1.1 strains, respectively); aa residues are 100% identical across all sub-genotypes in regions 2 (^267^ FFLTLKYGINT ^277^) and 3 (^322^ FAPAEYAQLYSFAMG ^336^). Regarding the P protein sequences, the RNA editing site (consensus motif: ^394^ AAAAAGGG ^401^) that regulates the generation of V and W proteins (17) is conserved across all the analyzed sequences. For the M protein sequences, the six immunodominant epitopes (M_IDE1-6_; each consisting of 15 aa residues) as reported in LaSota (62) showed high conservation in the M_IDE1_ (aa residues 40–54), but several substitutions are present in the other five epitopes, most of which are in M_IDE2_ (aa residues 71–85; Supplementary Figure S3). The nuclear localization signal (consensus motif: ^246^ DKKGKKVTFDKIEEKIRR ^263^) is also highly conserved across the sub-genotypes, except for substitution in the Tanzanian XIII.1.1 (D246N) and V.3 (I257L and E259R) strains.

The receptor-binding sites of the HN protein (*n* = 17; aa residues R174, I175, D198, K236, E258, Y299, Y317, R363, F364, E401, R416, S418, I466, R498, R516, Y526, and E547) are highly conserved across the sub-genotypes. Regarding the HN antigenic sites known for NDVs (63), three aa substitutions are present (sites 2 and 3) in three Tanzanian compared to other strains of the same sub-genotypes (Table 3); these are D569G in the VII.2 strains 2,152-IM184/19 and 2,110-K103/2/18 from Iringa and Dar es Salaam regions, and D287E in V.3 strain 2,110-K103/1/18 from Dar es Salaam.

**Table 3:** Comparative analysis of the amino acid residues in the antigenic sites of the hemagglutinin-neuraminidase (HN) protein of the Tanzanian NDVs identified in the current study (shown in italics) with other strains.

Sub-genotype	Strain	Site 1	Site 2				Site 3		Site 4			Site 12		Site 14			Site 23		
		345	513	514	521	569	263	287	321	333	356	494	516	347	350	353	193	194	201
	Consensus	P	R	V	S	D	R	D	K	K	K	D	R	E	Y	R	L	S	H
V.3	*OR230621/ck/TZ/Dar es Salaam/2110-K103/1/18*	·	·	*I*	·	*N*	·	*E*	·	*R*	·	*N*	·	·	·	·	·	·	·
	MK583011/ck/TZ/Mbeya/MT15/12	·	·	I	·	N	·	·	·	·	·	N	·	·	·	·	·	·	·
	MN685354/ck/KE/KE001/15	·	·	I	·	N	·	·	·	R	·	N	·	·	·	·	·	·	·
	MN685355/ck/KE/KE0811/16	·	·	I	·	N	·	·	·	R	·	N	·	K	·	·	·	·	·
	MW342790/ck/KE/Bomet/34 MB09/17	·	·	I	·	N	·	·	·	R	·	·	·	·	·	·	·	·	·
VII.2	*OR230617/ck/TZ/Morogoro/2014-E734/18*	·	·	·	·	·	·	·	·	·	·	·	·	·	·	·	·	·	·
	*OR230627/ck/TZ/Iringa/2152-IM184/19*	·	·	·	·	*G*	·	·	·	·	·	·	·	·	·	·	·	·	·
	*OR230628/ck/TZ/Morogoro/2159-ME50/19*	·	·	·	·	·	·	·	·	·	·	·	·	·	·	·	·	·	·
	*OR230618/ck/TZ/Morogoro/2015-E735/18*	·	·	·	·	·	·	·	·	·	·	·	·	·	·	·	·	·	·
	*OR230616/ck/TZ/Morogoro/2007-E713/18*	·	·	·	·	·	·	·	·	·	·	·	·	·	·	·	·	·	·
	*R230619/ck/TZ/Morogoro/2016-E736/18*	·	·	·	·	·	·	·	·	·	·	·	·	·	·	·	·	·	·
	*OR230620/ck/TZ/Morogoro/2017-E740/18*	·	·	·	·	·	·	·	·	·	·	·	·	·	·	·	·	·	·
	*OR230611/ck/TZ/Dar es Salaam/1996-B03/18*	·	·	·	·	·	·	·	·	·	·	·	·	·	·	·	·	·	·
	*OR230612/ck/TZ/Dar es Salaam/1997-B06/18*	·	·	·	·	·	·	·	·	·	·	·	·	·	·	·	·	·	·
	*OR230625/ck/TZ/Dar es Salaam/2144-BD117/19*	·	·	·	·	·	·	·	·	·	·	·	·	·	·	·	·	·	·
	*OR230622/ck/TZ/Dar es Salaam/2110-K103/2/18*	·	·	·	·	*G*	·	·	·	·	·	·	·	·	·	·	·	·	·
	*OR230626/ck/TZ/Arusha/2145-CA127/19*	·	·	·	·	·	·	·	·	·	·	·	·	·	·	·	·	·	·
	*OR230624/ck/TZ/Morogoro/2115-M581/18*	·	·	·	·	·	·	·	·	·	·	·	·	·	·	·	·	·	·
	JN986837/ck/NL/152608/93 (ancestral)	·	·	·	·	·	K	·	·	·	·	·	·	·	·	·	·	·	·
	KR074406/ck/MY/MB076/05	·	·	·	·	N	K	·	·	·	·	·	·	·	·	·	·	·	·
	HQ697255/ck/ID/Sukorejo/019/10	·	·	·	·	·	·	·	·	·	·	·	·	·	·	·	·	·	·
	KR074404/ck/MY/IBS002/11	·	·	·	·	·	·	·	·	·	·	·	·	·	·	·	·	·	·
	KR815908/tk/ZA/N2057/13	·	·	·	·	·	·	·	·	·	·	·	·	·	·	·	·	·	·
XIII.1.1	*OR230623/ck/TZ/Dar es Salaam/2111-K104/18*	·	·	·	·	*V*	*K*	·	·	·	·	·	·	*G*	·	·	·	·	·
	*OR230613/ck/TZ/Dar es Salaam/1998-B08/18*	·	·	·	·	*V*	*K*	·	·	·	·	·	·	*G*	·	·	·	·	·
	*OR230614/ck/TZ/Dar es Salaam/2000-B18/18*	·	·	·	·	*V*	*K*	·	·	·	·	·	·	*G*	·	·	·	·	·
	*OR230615/ck/TZ/Dar es Salaam/2001-B20/18*	·	·	·	·	*V*	*K*	·	·	·	·	·	·	*G*	·	·	·	·	·
	GU585905/ck/SE/97	·	·	·	·	A	K	·	·	·	·	·	Q	·	·	·	·	·	·
	MK633953/ck/TZ/Tanga/N34/12	·	·	·	·	V	K	·	·	·	·	·	·	G	·	·	·	·	·
	MK633935/ck/TZ/Iringa/IM40/12	·	·	·	·	V	K	·	·	·	·	·	·	G	·	·	·	·	·
	MK633944/ck/TZ/Arusha/AC53/12	·	·	·	·	V	K	·	·	·	·	·	·	G	·	·	·	·	·
	MK633933/ck/TZ/Mbeya/MT1/12	·	·	A	·	V	K	·	·	·	·	·	·	·	·	·	·	·	·
	KF727980/ck/IN/Bareilly/06	·	·	·	·	A	K	·	·	·	·	·	·	·	·	·	·	·	·

Dots (“·”) indicate identical residues. Amino acid substitutions in the Tanzanian strains compared to other strains are in bold and underlined.

The L protein sequence is largely conserved across the analyzed sequences of the same sub-genotypes, particularly in the functional domains such as the RNA-dependent RNA polymerase (RdRp) catalytic domain (aa residues 640–818) and mRNA-capping domain V (aa residues 1,112–1,355). There are however four aa substitutions in the RdRp catalytic domains of the Tanzanian VII.2 strains compared to other strains, including H741R and K783R substitutions (Supplementary Figure S4). The mRNA-capping domain contained six aa variations amongst strains of the same sub-genotypes, mostly in VII.2 strains.

#### 3.3.4. Phylogenetic analysis

**Figure 4:**
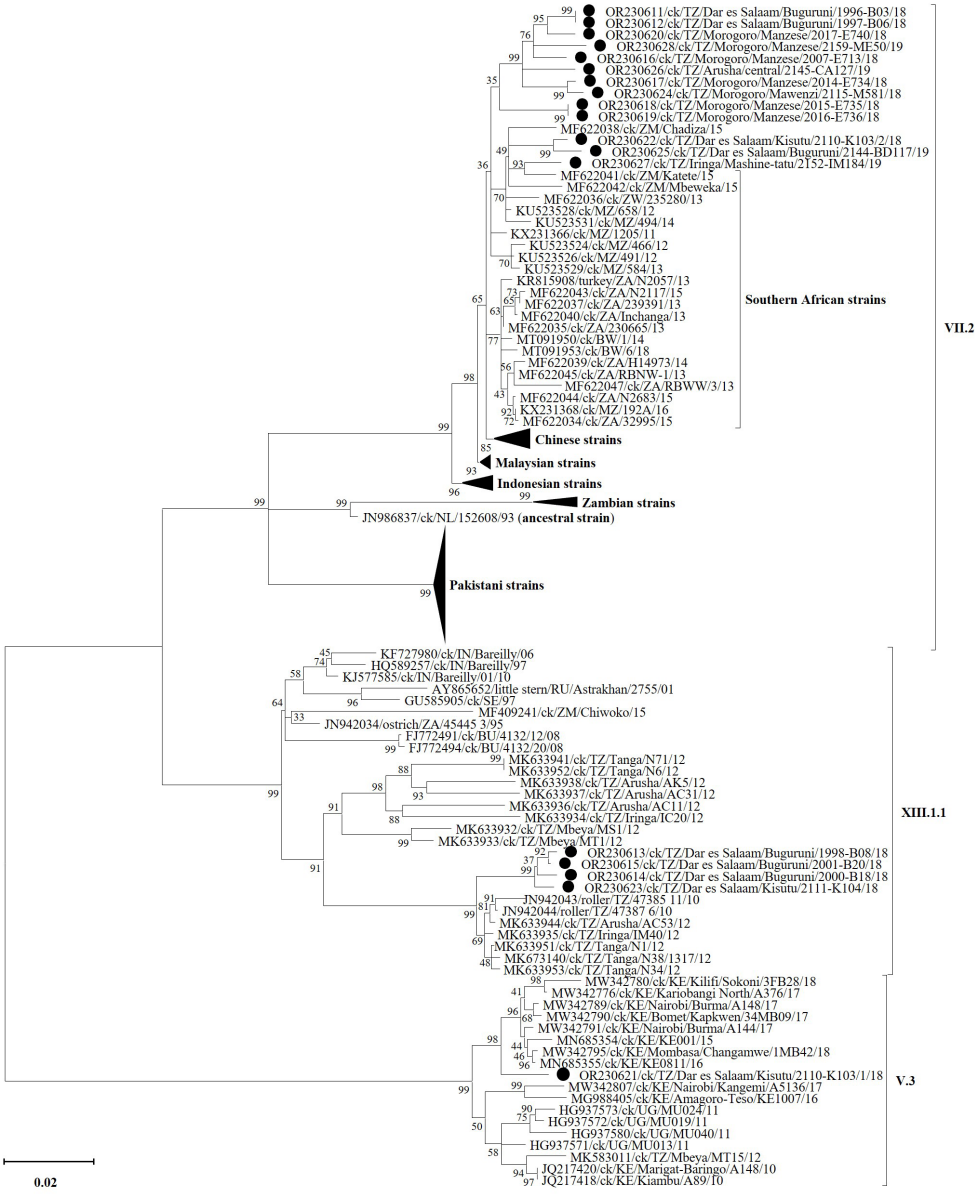
Phylogenetic analysis of the strains of NDV sub-genotypes V.3 (*n* = 1), VII.2 (*n* = 13), and XIII.1.1 (*n* = 4) identified in the current study (marked by black circles) and other strains based on the complete fusion (F) gene nucleotide sequences. Sequence names include GenBank accession numbers, bird species, 2-letter country abbreviation, sampling location, strain name and year of sample collection. The NDV (sub)-genotypes are based on the current updated unified nomenclature (64). The analysis was performed as described in the text with the final dataset consisting of 118 sequences and 1,662 positions (a detailed phylogenetic tree is presented in Supplementary Figure S1).

Complete F gene nucleotide sequences phylogenetically cluster the Tanzanian VII.2 strains from the current study with viruses previously reported from Mozambique, Zambia and Zimbabwe, which are Tanzania’s immediate southern neighbors (Figure 4; see detailed tree in Supplementary Figure S1). This cluster is distinct from that containing strains from the southernmost countries in the region (i.e., Botswana, Namibia and South Africa), as well as Chinese, Malaysian and Indonesian strains. All four Tanzanian XIII.1.1 strains from this study (all from Dar es Salaam region) group distinctly from the older (2010 and 2012) strains reported from Arusha, Iringa, Tanga and Mbeya (65). The single sub-genotype V.3 strain identified from Dar es Salaam region groups with, but distinctly from, the 2015–2018 Kenyan strains (29); this group is distinct from that containing the Tanzanian 2012 MT15 V.3 reference strain (30), the Kenyan 2010 and 2016–17 strains (31) and the 2011 Ugandan strains (32). Nucleotide sequences of all other five viral genes and the complete genomes cluster the Tanzanian strains from the current study distinctly from other viruses in their respective sub-genotypes, except for the M-gene sequences of sub-genotype VII.2 (Supplementary Figure S2).

### 3.4. Viruses coinfecting with NDVs

Table 4 shows the viral agents that coinfected with the NDVs and were detected in sufficient number of NGS reads to allow for assembly of complete gene coding sequences (CDS), of which IBV was the most abundant coinfecting virus being identified in seven out of nine chickens (see Supplementary Table S2). Two complete genome sequences of IBVs (27,686 and 27,663 nt in length) were assembled from OP samples of chickens swabbed at central and Buguruni LBMs in Arusha and Dar es Salaam regions, respectively (Table 4). Further analyses showed that one of the IBVs (strain 2,145-CA127; GenBank accession number OQ725698) is a recombinant virus with a backbone derived from an LX4-like IBV (lineage GI-19) virus and a turkey CoV-like S-gene; We have recently published the sequence data of this recombinant strain in a separate paper (66).

**Table 4:** NDV coinfecting viral agents for which complete genome or protein coding gene (CDS) could be assembled from the NGS of oropharyngeal (OP) or cloacal (CL) swabs.

Chicken ID	Region (LBM location)	Sample type	Viral agent	Virus-specific-reads	Genome assembly				Genotype; genogroup	GenBank accession number
					Genomic region	consensus seq length	Median cov. depth (reads)	Completeness (%)		
1995-B01	Dar es Salaam (Buguruni)	OP	IBV	80,135	Complete genome	27,686	758	99.92%	GI-19	OR180678
1998-B08	Dar es Salaam (Buguruni)	CL	IBV	142,023	Genes 3a/b, E, M, 4b/c, 5a/b, N and 6b	3,827	451	100.0%	N/A	OR230609
2110-K103	Dar es Salaam (Kisutu)	CL	IBV	164,536	genes 3a/b, E, M, 4b/c, 5a/b, N and 6b	3,673	652	100.0%	N/A	OR230610
2145-CA127	Arusha (Central)	OP	IBV	29,470	Complete genome	27,663	262	98.50%	GI-19 (TCoV-like) recombinant	OQ725698
2151-IM162	Iringa (Miomboni)	CL	CAstV	9,392	Complete genome	6,938	1,502	100.0%	CAstV-Aii	OQ685945
			ANV	15,643	Complete genome	7,318	479	100.0%	ANV-5	OQ685946

ANV, avian nephritis virus; CAstV, chicken astrovirus; IBV, infectious bronchitis virus.

In addition to the IBVs, complete genome sequences of an enteric chicken astrovirus (CAstV; 7,318-nt in length) and an avian nephritis virus (ANV; 6,918-nt in length), the two type species of genus *Avastrovirus* (AAstV), were assembled from the CL sample of chicken ID 2151-IM162 (Table 4). The two genomes have the typical AAstV architecture (5′-UTR-ORF1a-ORF1b-ORF2-′3-UTR) and classify with Eurasian CAstV antigenic group Aii (CAstV-Aii) and ANV group 5 (ANV-5) with the CAstV-Aii being a recombinant of CAstV-Bi and Bvi. We have recently published the genomic data of the two Tanzanian AAstVs in a separate paper (67).

## 4. Discussion

Small-scale backyard chicken rearing is an integral part of rural communities and contributes an estimated 37–40% of income-generating household activities in Tanzania (37, 68). The country has an estimated backyard chicken population of about 38.8 million (~46.55% of the total chicken population), which are kept by about 78% of rural agricultural households (69, 70). Like in many developing countries, the main challenge to backyard chicken production in Tanzania is infectious diseases, of which NDV has the greatest impact by causing abnormally high mortalities in unvaccinated flocks (33, 71). The high mobility of these flocks during their daily scavenging for food, and their interactions with neighboring village poultry flocks and wild bird species could significantly contribute to the viral persistence and transmission as has been recently demonstrated (72). Furthermore, Tanzania is not any different from other sub-Saharan Africa countries where disease surveillance, diagnostics and vaccination of rural poultry are the exception rather than the rule; most farmers sell off their flocks in their neighborhoods, or use ineffective traditional methods at the onset of clinical disease signs, and some of them may be unaware of vaccination as a disease control option due to constrained veterinary extension services (37). Only apparently healthy but potentially subclinically infected chickens are presented for sale at the informal LMBs in urban settlements. The high density congregation of chickens from various regions at the LBMs is ideal for viral replication, persistence and rapid spread, especially in cases where some of the chickens bought at the markets are taken back to the villages as seed stocks (33). Considering the above scenarios, the surveillance of avian viruses such as presented in the current study are necessary for understanding the viral epidemiology in rural poultry.

The present study was designed to survey (identify and molecularly characterize) NDVs circulating in backyard chickens traded at the major LBMs in Dar es Salaam, Morogoro, Tanga, Arusha, Iringa and Mbeya regions of Tanzania. Our results demonstrate higher NDV positivity rates in the chickens from LMBs located in the east-coast (42.91% in Dar es Salaam), mid-eastern (31.99% in Morogoro) and north-eastern (30.26% in Tanga) regions of Tanzania compared to birds from the south-western (16.78% in Mbeya) and central (8.09% in Iringa) regions of the country. The observed higher positivity rates during the dry season compared to the rainy season in the current study have been reported elsewhere in Africa (73–75). The detection of NDVs in both OP and CL samples of ~38% of the birds implicates active viral shedding, and the observed higher amounts of viral RNAs in the OP samples compared to their CL counterparts supports recent infections before the chickens become clinical as evidenced by other studies (76). This may however be an underrepresentation of the actual proportions of birds potentially shedding viruses at the time of sampling because only about 38% of the study chickens had both their OP and CL rRT-PCR-tested. There were no records of observable clinical signs of disease during sampling, mainly because unhealthy-looking birds are usually quickly slaughtered before further progression of overt clinical signs of disease (33). Nevertheless, apparently healthy but subclinically (or latently) infected or recovered birds are reservoirs for NDV transmission to naïve susceptible birds (77).

Consistent with the observed trend in the NDV positivity rates, most of the complete genome sequences were assembled from the chickens swabbed from the east-coast Dar es Salaam region (the single V.3 strain, four of the 13 VII.2 strains and all four XIII.1.1 strains) and the mid-eastern Morogoro region (seven of the 13 VII.2 strains). Furthermore, the two instances of chickens with mixed NDV infections were both from the Dar es Salaam LMBs in Kisutu (V.3/VII.2) and Buguruni (VII.2/XII.1.1), and four of the nine instances of coinfections were found in chickens from Buguruni. Notably, the Buguruni and Kisutu LBMs are the largest in Tanzania where chickens from regions outside of the Dar es Salaam are traded, which is unlike in the other LBMs where the birds are typically sourced from villages in close proximity to the markets within the regions (33). One would logically expect the two Dar es Salaam LBMs to be richer in the diversity of NDVs and other avian viruses compared to LBMs in other regions. Indeed, our data strongly suggest this to be the case as evidenced by the phylogenetic clustering of the four Dar es Salaam VII.2 strains into two groups. One group contains strains 1996-B03 and 1997-B06 from Buguruni LBM that monophyletically cluster with, but distinctly from the seven Morogoro strains and strain 2,145-CA127 from Arusha region in the northern Tanzania/Kenya border. The other group containing strains 2,110-K103 and 2,144-BD117 (Kisutu and Buguruni LBMs) cluster separately with strain 2,152-IM184 from Iringa region (south of Tanzania) and the 2015 strains from Tanzania’s southern neighbors (Zambia and Mozambique). The only other sub-type VII virus from Tanzania is a partial F-gene sequence (786 bp in length; GenBank accession: MT335749), which was reported in 2012 from Mwanza region (78). Similarly, all four XIII.1.1 strains identified in the current study (three strains from Buguruni and one strain from Kisutu LBMs) cluster distinctly from all the older (2010–2012) Tanzanian strains; the Kisutu strain 2,111-K104 appears distinct from the Buguruni strains with high bootstrap support (99%). Furthermore, the single V.3 strain identified from Kisutu LMB clusters with, but distinctly from the Kenyan strains reported between 2015 and 2018, away from the only other complete genome sequence of V.3 reported from Tanzania in 2012 [strain MT15; GenBank accession: MK583011 (30)], and the Ugandan 2011 strains (32). To the best of our knowledge, sub-genotype V.3 viruses are yet to be reported outside of the three East African countries (Kenya, Uganda and Tanzania). Several F-gene partial sequences (785–790 bp in length) of V.3 viruses have been reported from Morogoro region (78). Overall, the Tanzanian NDVs are distinct from strains reported from the eastern and southern Africa regions.

Consistent with the WOAH criteria for virulence (6), we have demonstrated (by the confirmatory rRT-PCR F-test, sequence analyses and ICPI testing) that the Tanzanian V.3, VII.2 and XIII.1.1 strains reported in the current study are vNDVs (F_1_/F_2_ cleavage with the consensus motifs ^112^ RRRKR↓F ^117^ and ^112^ RRQKR↓F ^117^). This finding is not surprising because ND is endemic in the country (33), but the identification of vNDVs in apparently healthy chickens traded at LBMs in four out of the six study regions (Dar es Salaam, Morogoro, Arusha and Iringa) is notable because it implicates their wide distribution in the country, and most likely in the larger east African region. Coinfections of the same bird with viral and/or bacterial pathogens such as that of the vNDVs in the current study are likely to result in alterations of their pathogenicity, compromise the host immune system and complicate their diagnostics and control due to difficulties in distinguishing clinical manifestations of the disease they cause in infected poultry.

## 5. Conclusion

This study has demonstrated that the positivity of the NDVs is higher in the chickens at the LBMs in the east coast Dar es Salaam region where traders source their birds from various regions in Tanzania, and higher during the dry compared to the rainy season. We have also demonstrated that the V.3, VII.2, and XIII.1.1 NDVs from backyard chickens in Tanzania are virulent and that they phylogenetically cluster regionally in the country and distinctly from the strains previously reported in the eastern and southern Africa countries. Additionally, we found that some of the birds had mixed infections with different vNDV genotypes and/or coinfected with other viruses, most notable being IBV and AAstV strains, some of which are recombinant viruses. Considering the high NDV-positive rate by the rRT-PCR test, it is likely that vNDVs are wildly distributed in the country, with the further possibility of their transmission across the neighboring countries through the highly unregulated live poultry trade between the countries in the eastern and southern African region. Our data add to the repertoire of NDV sequence data that are bound to be useful for further investigations into NDV and other coinfecting viral and bacterial pathogens.

## Data availability statement

The datasets presented in this study can be found in online repositories. The names of the repository/repositories and accession number(s) can be found at: https://www.ncbi.nlm.nih.gov/genbank/, OR230611-OR230628; https://www.ncbi.nlm.nih.gov/bioproject/PRJNA945007, SRR25147074-SRR25147106.

## Ethics statement

The animal study was approved by The National Poultry Research Center Institutional Animal Care and Use Committee (IACUC). The study was conducted in accordance with the local legislation and institutional requirements.

## Author contributions

HK: Conceptualization, Data curation, Formal analysis, Investigation, Methodology, Validation, Visualization, Writing – original draft, Writing – review & editing. JV: Data curation, Formal analysis, Methodology, Software, Validation, Visualization, Writing – review & editing. GC: Methodology, Writing – review & editing. IG: Data curation, Investigation, Methodology, Writing – review & editing. TO: Investigation, Methodology, Writing – review & editing. PM: Conceptualization, Funding acquisition, Project administration, Resources, Supervision, Writing – review & editing. DS: Conceptualization, Funding acquisition, Project administration, Resources, Supervision, Writing – review & editing.

## Footnotes

^1^http://www.rstudio.com

^2^github.com/FelixKrueger/TrimGalore

^3^https://sourceforge.net/projects/bbmap/

^4^www.geneious.com
