# Supplementary material for: Virulent Newcastle disease virus genotypes V.3, VII.2, and XIII.1.1 and their coinfections with infectious bronchitis viruses and other avian pathogens in backyard chickens in Tanzania
Source: Front Vet Sci. 2023 Oct 19;10:1272402. doi: 10.3389/fvets.2023.1272402 (PMC10625407; doi:10.3389/fvets.2023.1272402)
Supplement: Supplementary file 1 [file Data_Sheet_1.docx]

Supplementary Material

Table S1. Summary of backyard chickens whose both OP and CL swab samples were screened for NDV using the rRT-PCR L-test in the current study. The samples were collected from 12 live bird markets (LMBs) in six regions in Tanzania. Samples with rRT-PCR cycle threshold (C_T_) values of ≤ 40 and ≤ 25 were considered to be “likely” and “strongly” NDV-positive, respectively.

| **Region** | **LBM location** | **rRT-PCR L-test results on both OP and CL samples** | | | |
| --- | --- | --- | --- | --- | --- |
|  |  | **total birds tested** | **total likely NDV-positive birds (*C*_T_ <40)** | **birds with high amounts of viral RNAs (*C*_T_ <25)** | |
|  |  |  |  | **OP** | **CL** |
| Arusha | central | 75 | 37 | 5 | 9 |
|  | Kilombero | 0 | 0 | 0 | 0 |
| Dar es Salaam | Buguruni | 99 | 50 | 14 | 5 |
|  | Kisutu | 2 | 2 | 2 | 1 |
| Iringa | Mashine tatu | 68 | 5 | 3 | 1 |
|  | Miomboni | 8 | 1 | 1 | 0 |
| Mbeya | Sokomatola | 0 | 0 | 0 | 0 |
|  | Soweto | 17 | 4 | 0 | 0 |
| Morogoro | Manzese | 6 | 6 | 6 | 1 |
|  | Mawenzi | 1 | 1 | 1 | 1 |
| Tanga | Ngamiani | 0 | 0 | 0 | 0 |
|  | Uzunguni | 0 | 0 | 0 | 0 |
| **Total** | | **276** | **106 (38.41%)** | **32 (30.48%)** | **18 (17.14%)** |

Table S2. Overview of the results obtained from rRT-PCR NDV detection and NGS NDVs in oropharyngeal (OP), cloacal (CL) and allantoic fluid (AF) samples from 20 backyard chickens, which were selected as described in the text. OP samples from nine out of the 20 birds were randomly selected for intracerebral pathogenicity index (ICPI) test as described in the text. For each sample, microbial agents (viral and bacterial) of avian interest other than the NDVs are indicated, i.e., agents that are known to cause and/or are associated with avian disease based on available literature and expert knowledge. The number of NGS reads obtained for each of taxon are shown in brackets. The table only shows microbial agents identified with high confidence based on heuristic cutoffs developed by BASE₂BIO LLC to eliminate false positives (minimum read count 50, minimum k-mer count 200).

| **Chicken ID** | **Collection date** | **Region (LBM location)** | **Sample type** | **NDV rRT-PCR (*C*_T_ value)** | | | **NGS read count** | | | **Other identified agents of avian interest (number of taxon-specific NGS reads identified with)** | |
| --- | --- | --- | --- | --- | --- | --- | --- | --- | --- | --- | --- |
|  |  |  |  | **L-test** | **M-test** | **F-test** | **Total** | **Host-specific** | **NDV-specific** | **Viral** | **Bacterial** |
| 2014-E734 | 10-Oct-18 | Morogoro (Manzese) | OP | 16.54 | 31.97 | 26.53 | 542,621 | 12.70% | 84,480 | - | *E. faecium* (1198); *M. synoviae* (1152); *Campylobacter* (189); *Avibacterium* (425) |
|  |  |  | CL | 37.12 | negative | negative | 487,868 | 32.45% | - | - | - |
| 2110-K103 | 22-Sep-18 | Dar es Salaam (Kisutu) | OP | 16.37 | negative | 22.68 | 535,135 | 21.13% | 86,610 | IAV H5 (50) | *E. faecium* (972); *Avibacterium* (1014); ORT (258) |
|  |  |  | CL | 26.32 | 28.56 | 23.90 | 534,566 | 20.12% | 11,764 | IBV (164,536); CAstV (198) | *E. faecium* (434) |
| 2111-K104 | 22-Sep-18 | Dar es Salaam (Kisutu) | OP | 16.37 | 23.04 | 29.19 | 517,246 | 5.46% | 375,478 | - | *E. faecium* (201); *Avibacterium* (200); ORT (848); *M. synoviae* (198); *Campylobacter* (80) |
|  |  |  | CL | 24.90 | 30.47 | 38.26 | 570,124 | 40.99% | 12,113 | - | *E. faecium* (716); *Avibacterium* (1482); *Gallibacterium* (160) |
| 2115-M581 | 5-Oct-18 | Morogoro (Mawenzi) | OP | 17.80 | 23.41 | 25.96 | 344,285 | 13.63% | 143,124 | - | *E. faecium* (1153); *Avibacterium* (777): *M. synoviae* (442); *Campylobacter* (62) |
|  |  |  | CL | 21.12 | 22.75 | 25.84 | 606,314 | 1.81% | 4488 | - | - |
| 2144-BD117 | 24-May-19 | Dar es Salaam (Buguruni) | OP | 16.82 | 22.38 | 36.69 | 373,719 | 35.14% | 32,728 | - | *E. faecium* (318); *Avibacterium* (825); *Campylobacter* (1751); *Gallibacterium* (129) |
|  |  |  | CL | 36.49 | negative | negative | 763,677 | 31.32% | - | - | *E. faecium* (3768); *Avibacterium* (1366); *Campylobacter* (840); *Gallibacterium* (140) |
| 2145-CA127 | 3-May-19 | Arusha (Central) | OP | 16.79 | 19.23 | 24.19 | 510,552 | 7.80% | 177,140 | IBV (29,470); ChMeV (110) | *E. faecium* (202); ORT (1638); *M. synoviae* (953); *Avibacterium* (303) |
|  |  |  | CL | 28.65 | 27.32 | 37.12 | 633,437 | 46.28% | 618 | IBV (281) | *E. faecium* (270); *Avibacterium* (230) |
| 2151-IM162 | 11-Apr-19 | Iringa (Miomboni) | OP | 24.23 | 28.27 | negative | 551,912 | 20.30% | 1208 | - | *E. faecium* (701); *Avibacterium* (762); |
|  |  |  | CL | negative | negative | negative | 588,771 | 9.40% | - | CAstV (9392); ANV (15,643); SiV (1688) | *E.* *cecorum* (1141); *E. faecium* (458); *Avibacterium* (266); *Gallibacterium* (297) |
| 2152-IM184 * | 11-Apr-19 | Iringa (Mashine tatu) | OP | 16.45 | 21.42 | 31.48 | 545,191 | 17.36% | 107,852 | IBV (316) | *E. faecium* (448); *Avibacterium* (443); ORT (60); *M. gallisepticum* (71) |
| 2159-ME50 | 29-May-19 | Morogoro (Manzese) | OP | 14.25 | 22.28 | 28.53 | 653,798 | 17.33% | 63,407 | - | *Avibacterium* (659); *E. faecium* (89); *Campylobacter* (1574); ORT (1209) |
|  |  |  | CL | 29.23 | 29.31 | 35.47 | 575,850 | 34.12% | 1860 | - | *E. faecium* (933); *Avibacterium* (596) |
| 1995-B01 | 21-Sep-18 | Dar es Salaam (Buguruni) | OP | 14.52 | 16.98 | 28.37 | 375,997 | 5.14% | 173,620 | IBV (80,135) | ORT (34,244); *Campylobacter* (1951) |
|  |  |  | CL | 27.94 | 27.96 | 37.38 | 349,605 | 2.21% | - | IBV (11,119) | *Campylobacter* (8421) |
|  |  |  | AF | 18.55 | 17.39 | 26.59 | 406,279 | 3.69% | 73,140 | IBV (66,533) | - |
| 1996-B03 | 21-Sep-18 | Dar es Salaam (Buguruni) | OP | 19.29 | 23.24 | 34.47 | 314,404 | 10.16% | 16,823 | - | *Avibacterium* (358); ORT (657); *Campylobacter* (284) |
|  |  |  | CL | 20.95 | 21.14 | 32.70 | 343,702 | 9.56% | 106,015 | - | - |
|  |  |  | AF | 15.80 | 16.00 | 25.91 | 400,351 | 1.65% | 390,295 | - | - |
| 1997-B06 | 21-Sep-18 | Dar es Salaam (Buguruni) | OP | 23.39 | 25.70 | 38.00 | 345,113 | 13.22% | 8625 | - | *Avibacterium* (338); ORT (740) |
|  |  |  | CL | 20.45 | 20.73 | 32.42 | 263,857 | 15.93% | 168,371 | - | - |
|  |  |  | AF | 16.99 | 17.52 | 28.09 | 391,413 | 1.92% | 378,390 | - | - |
| 1998-B08 | 21-Sep-18 | Dar es Salaam (Buguruni) | OP | 24.04 | 32.08 | 33.50 | 326,010 | 9.27% | 2572 | IBV (19,814); ChMeV (264) | *Campylobacter* (1,545); *Avibacterium* (201); ORT (105) |
|  |  |  | CL | 37.12 | negative | negative | 333,537 | 22.75% | - | IBV (142,023) | *Gallibacterium* (393) |
|  |  |  | AF | 14.92 | 17.84 | 20.73 | 481,272 | 1.16% | 471,551 | - | - |
| 1999-B09 | 21-Sep-18 | Dar es Salaam (Buguruni) | OP | 23.97 | 32.54 | 35.85 | 318,573 | 7.57% | 895 | IBV (11,847); AvRV-G (1028) | *Campylobacter* (4159); *M. gallisepticum* (3690); *M. synoviae* (208); *Avibacterium* (217); ORT (538) |
|  |  |  | CL | negative | negative | negative | 386,641 | 1.38% | - | - | - |
|  |  |  | AF | 16.38 | 19.31 | 22.24 | 494,234 | 2.14% | 123,204 | IBV (102,468) | - |
| 2000-B18 | 21-Sep-18 | Dar es Salaam (Buguruni) | OP | 19.56 | 26.56 | 33.45 | 258,751 | 18.08% | 92,145 | - | *E. faecium* (506); *Avibacterium* (531); *M. gallisepticum* (575) |
|  |  |  | CL | 26.92 | 31.11 | 36.44 | 521,509 | 66.64% | 372 | - | *Campylobacter* (382) |
|  |  |  | AF | 14.89 | 18.34 | 25.56 | 399,283 | 0.86% | 392,000 | - | - |
| 2001-B20 | 21-Sep-18 | Dar es Salaam (Buguruni) | OP | 18.88 | 25.98 | 28.58 | 343,919 | 6.43% | 55,631 | - | *Campylobacter* (1,951); ORT (743); *Gallibacterium* (468); *Avibacterium* (411) |
|  |  |  | CL | 23.58 | 27.47 | 29.67 | 425,170 | 14.46% | 12,063 | - | *E. faecium* (968); *Avibacterium* (1270); *Gallibacterium* (407); *Campylobacter* (232) |
|  |  |  | AF | 17.13 | 19.83 | 20.86 | 419,443 | 0.05% | 16,849 | - | - |
| 2007-E713 | 10-Oct-18 | Morogoro (Manzese) | OP | 16.14 | 20.46 | 24.42 | 335,278 | 28.92% | 37,842 | ChMeV (116) | *E. faecium* (239); *M. synoviae* (157) |
|  |  |  | CL | 29.88 | 28.78 | 33.20 | 428,768 | 6.41% | - | IBV (964) | - |
|  |  |  | AF | 13.98 | 12.42 | 17.04 | 373,022 | 0.90% | 213,901 | - | - |
| 2015-E735 | 10-Oct-18 | Morogoro (Manzese) | OP | 24.80 | 25.00 | 32.30 | 348,489 | 27.94% | 6392 | - | *E. faecium* (1,081); *Avibacterium* (1,084); *M. synoviae* (794); *Campylobacter* (1708) |
|  |  |  | CL | 31.94 | 29.04 | 34.54 | 393,884 | 31.89% | - | - | *E. faecium* (930); *Avibacterium* (553); *Campylobacter* (122) |
|  |  |  | AF | 15.71 | 12.83 | 18.66 | 435,915 | 1.65% | 425,238 | - | *-* |
| 2016-E736 | 10-Oct-18 | Morogoro (Manzese) | OP | 17.06 | negative | 24.57 | 298,118 | 16.91% | 107,516 | aMPV-B (108) | *E. faecium* (325); *M. synoviae* (957); *M. gallisepticum* (191); *Avibacterium* (297) |
|  |  |  | CL | 25.89 | 22.62 | 27.65 | 349,910 | 7.83% | 1612 | - | *Campylobacter* (1800); *Avibacterium* (112) |
|  |  |  | AF | 16.62 | 13.45 | 19.31 | 357,373 | 1.14% | 350,402 | - | - |
| 2017-E740 | 10-Oct-18 | Morogoro (Manzese) | OP | 14.79 | negative | 32.72 | 384,455 | 11.07% | 214,867 | - | *E. faecium* (288); *Avibacterium* (222); *M. synoviae* (137); *Campylobacter* (266) |
|  |  |  | CL | 17.79 | negative | 29.64 | 387,171 | 14.76% | 302,084 | - | - |
|  |  |  | AF | 15.11 | negative | 29.00 | 563,061 | 5.86% | 463,798 | - | - |

The CL sample for chicken marked with asterisk (“*”; ID 2152-IM184) was unavailable.

**Abbreviations**: aMPV, avian metapneumovirus; ANV, avian nephritis virus; AvRV, avian rotavirus; CAstV, chicken astrovirus; ChMeV, chicken megrivirus; *E. faecium/cecorum*; *Enterococcus faecium*/*cecorum*; IAV, Influenza A virus; IBV, infectious bronchitis virus; *M. gallisepticum*/*M. synoviae*, *Mycoplasma gallisepticum*/*synoviae*; ORT, *Ornithobacterium* *rhinotracheale*; RSV, Rous sarcoma virus; SiV, sicinivirus.


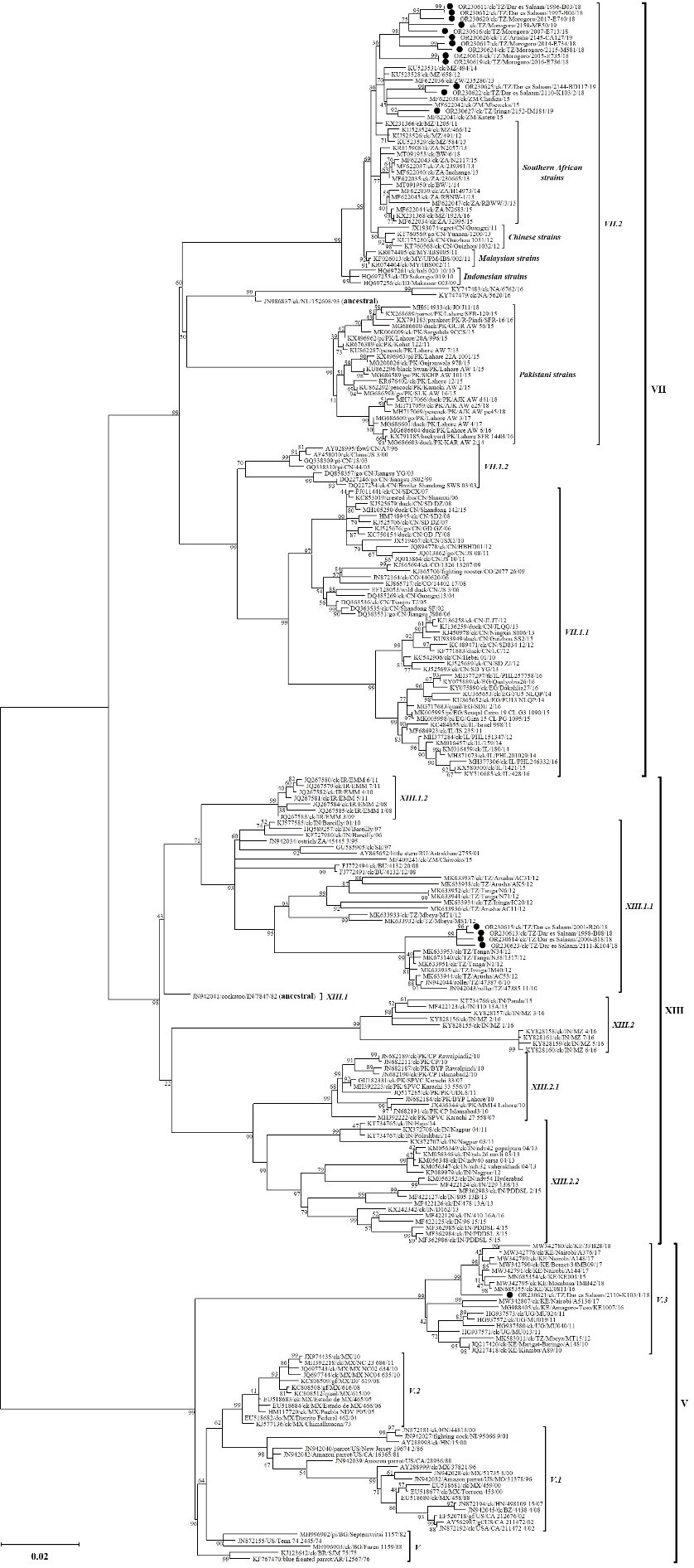


**FIG. S 1. Phylogenetic analyses of the strains of NDV sub-genotypes V.3 (n=1), VII.2 (n=13) and XIII.1.1 (n=4) identified in the current study (marked by black circles) and other strains based on the complete fusion (F) gene nucleotide sequences**. Sequence names include GenBank accession numbers, bird species, 2-letter country abbreviation, sampling location, strain name and year of sample collection. The NDV (sub)-genotypes are based on the current updated unified nomenclature (1). The analysis was performed as described in the text with the final dataset consisting of 254 sequences and 1662 positions.

.


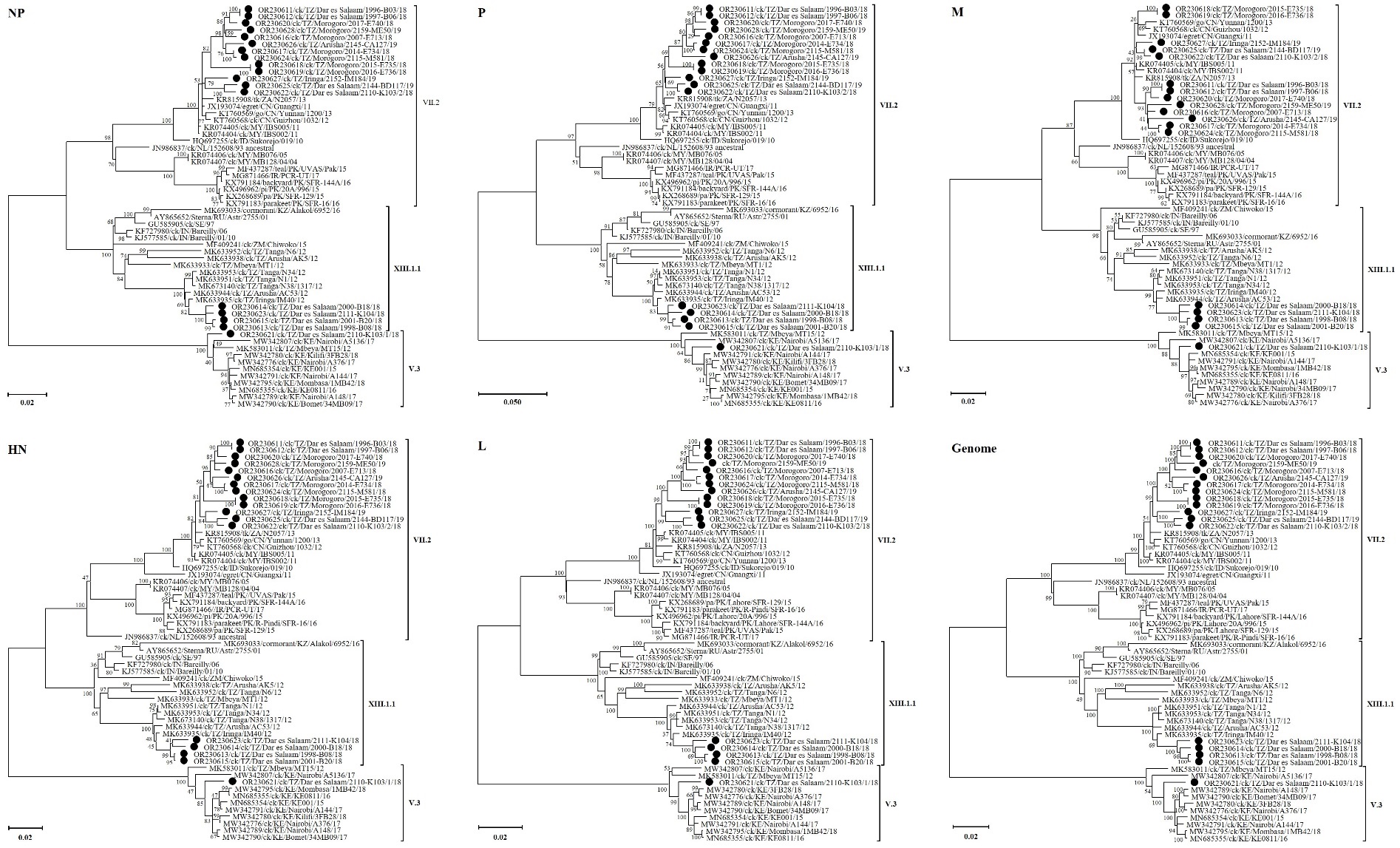


FIG. S 2. Phylogenetic analyses of the strains of NDV sub-genotypes V.3 (n=1), VII.2 (n=13) and XIII.1.1 (n=4) identified in the current study (marked by black circles) and other strains based on the complete nucleotide sequences of the nucleocapsid protein (NP), phosphoprotein (P), matrix (M), haemagglutinin-neuraminidase (HN), large polymerase (L) genes, and complete genome. Sequence names include GenBank accession numbers, bird species, 2-letter country abbreviation, sampling location, strain name and year of sample collection. The NDV (sub)-genotypes are based on the current updated unified nomenclature (1). The analysis was performed as described in the text with the final dataset involving 58 sequences and nucleotide positions as follows: 1470 (NP), 1188 (P), 1095 (M), 1716 (HN), 6615 (L), 15,051 (complete genome).


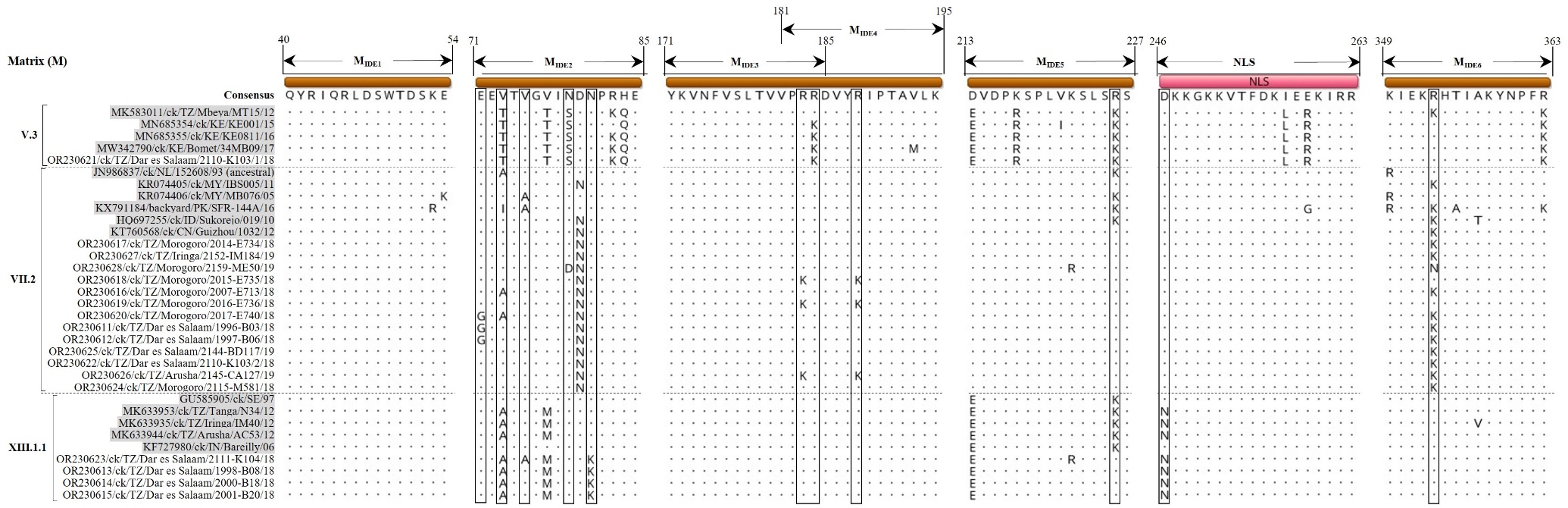


FIG. S 3. Conservation of amino acid residues in the immunodominant epitopes (MIDE1-6) and nuclear localization signal sequence (NLS) of the matric (M) protein sequences in the Tanzanian compared to a selection of other V.3, VII.2 and XIII.1.1 strains (shaded in gray color). Residues in the consensus sequence are numbers relative to the first methionine residue (M1) of the translated protein sequences. The vertical dotted lines separate the sub-genotypes. Open boxes indicate antibody neutralizing epitope region variations in the aa residues when comparing Tanzanian and other strains of the same sub-genotypes. Dots indicate identical aa residues.


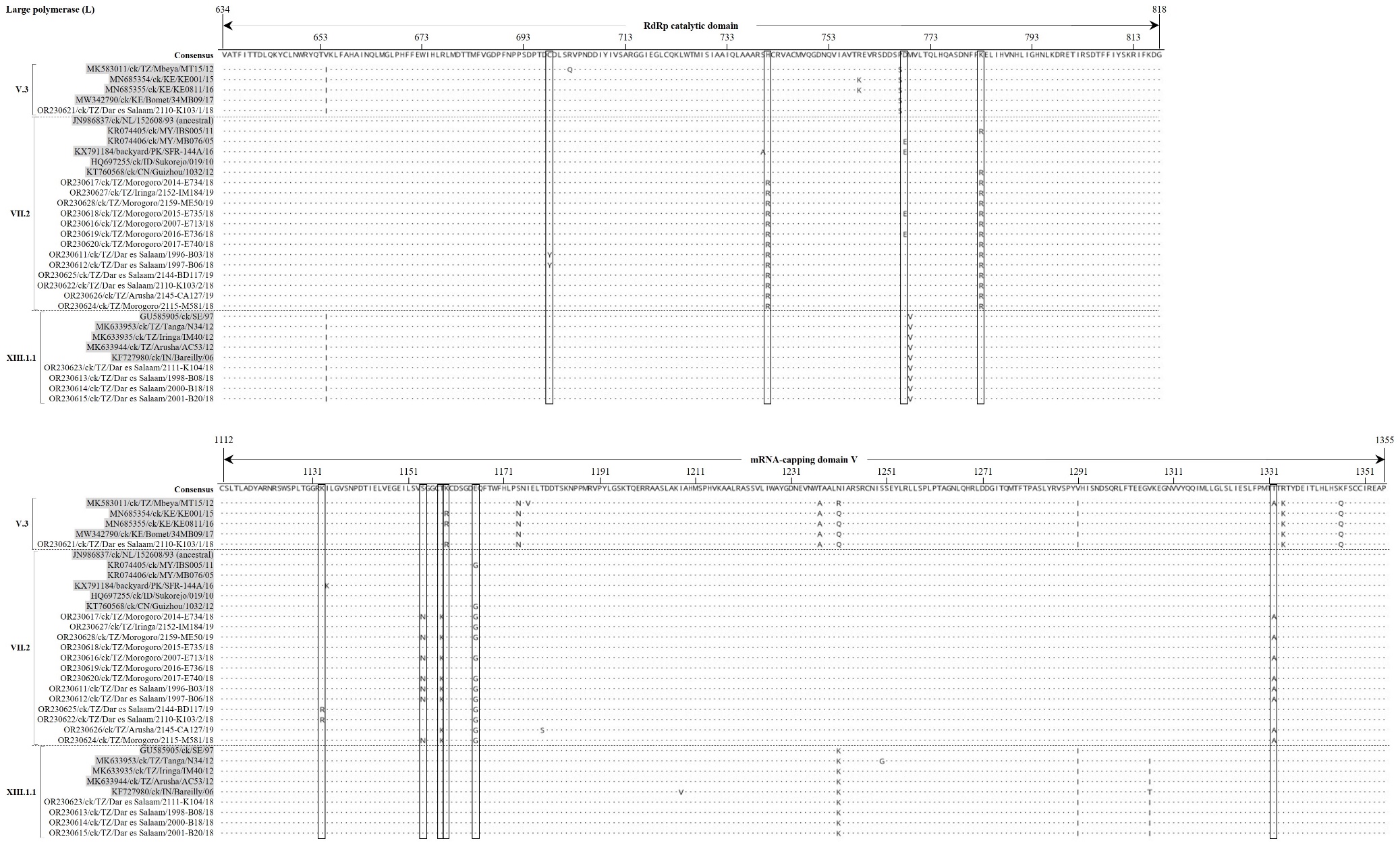


FIG. S 4. Conservation of amino acid residues in the RNA-dependent RNA polymerase (RdRp) catalytic domain and mRNA-capping domain V in the Tanzanian compared to a selection of other V.3, VII.2 and XIII.1.1 strains(shaded in gray color). Residues in the consensus sequence are numbers relative to the first methionine residue (M1) of the translated protein. Variations in the aa residues amongst the strains of different sub-genotypes are shown in open boxes. The vertical dotted lines separate the sub-genotypes. Dots indicate identical aa residues.


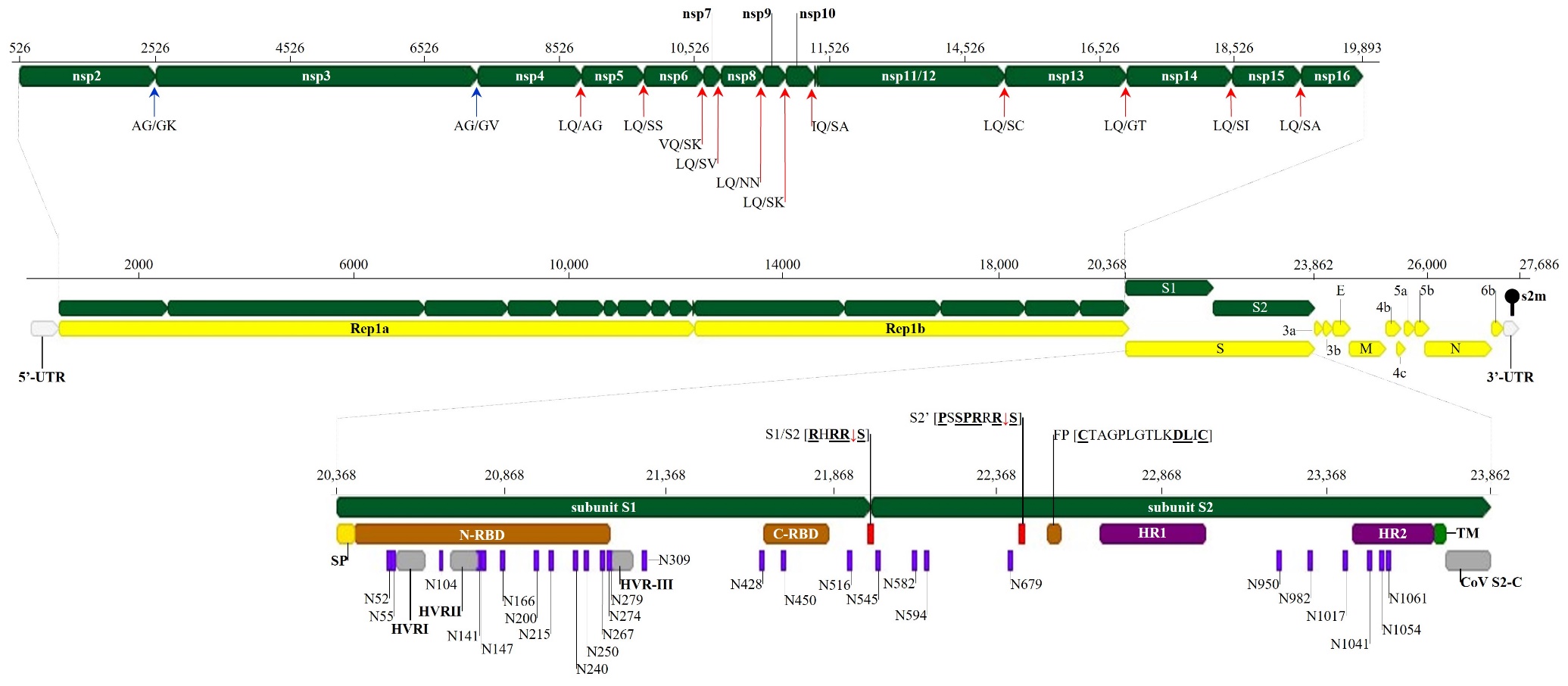


FIG. S 5. Schematic representation of genome features of the Tanzanian IBV strain 1995-B01 identified in the current study. The top panel shows the genomic (nucleotide) positions of the mature peptides of ORF1ab (replicase 1ab complex components; Rep1ab) and the consensus sequences of the proteolytic cleavage sites. Eleven of the 13 cleavage sites (shown in red arrow arrows are highly conserved in AvCoVs (consensus sequence x-[L/I/V/F/M]-Q↓[A/S/G], where “x” is any residue and “↓” is the cleavage site (2)). The bottom panel shows the structural featured of the spike glycoprotein. Features of subunit S1 include the N-terminal signal peptide (SP), N-and C-terminal receptor-binding domains (N-/C-RBD), and hypervariable regions (HVR I-III). The S1/S2 cleavage site is shown with the canonical motif of AvCoVs (R-X-[K/R]-R↓S, where “X” is any amino acid residue, conserved residues are in bold and underlined, and “↓” indicates cleavage position). Features found in the subunit S2 include auxiliary S2′ cleavage site (consensus motif P-X-S-P-R-X-R↓S (3)), fusion peptide (FP; consensus motif CTAGPLGTLKDLIC), heptad repeat regions (HR1 and HR2), transmembrane/non-cytoplasmic domain (TM), CoV C-terminal cysteine-rich intravirion region (CoV-S2-C), and *N*-linked glycosylation sites (*n*=27; shown in purple color and numbered using aa positions relative to the first methionine (M) residue of the S protein).


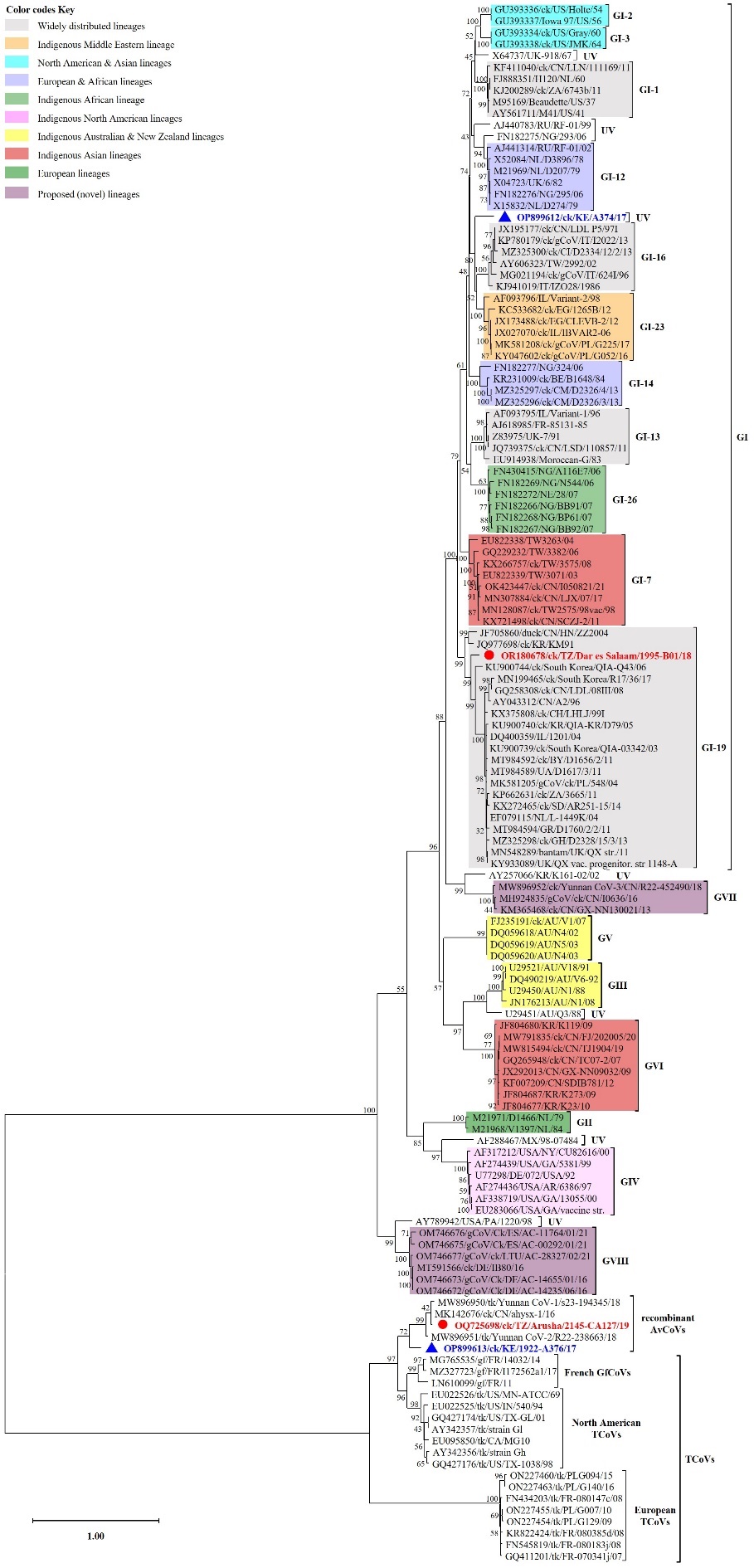


**FIG. S 6. Phylogenetic relationship of the infectious bronchitis viruses (IBVs) identified in the current study with other strains based on nucleotide sequences of the full-length S1 subunit.** The two Tanzanian strains and the recently reported strains from Kenya (Tanzanian northern neighbor) are shown in red and blue bold texts, respectively. The naming and the color-coded geographical distribution of the lineages are based on the current IBV classification system (4). Sequence names include GenBank accession numbers, abbreviated host species, country code, strain name and year of sample collection. The analysis was performed as described in the text with the final dataset consisting of 135 sequences and 1375 positions. Abbreviations: AvCoV, avian coronavirus; GI-VII, genotypes I to VIII; GfCoV, guinea fowl coronavirus; TCoV, turkey coronavirus; UV, unique variant.

# References

1. Dimitrov KM, Abolnik C, Afonso CL, Albina E, Bahl J, Berg M, Briand F-X, Brown IH, Choi K-S, Chvala I. Updated unified phylogenetic classification system and revised nomenclature for Newcastle disease virus. *Infect Genet Evol* (2019) 74:103917–32. doi: 10.1016/j.meegid.2019.103917

2. Snijder EJ, Decroly E, Ziebuhr J. “CHAPTER 3. The Nonstructural Proteins Directing Coronavirus RNA Synthesis and Processing.,” In: Ziebuhr J, editor. *Advances in Virus Research*. Cambridge, MA, USA: Academic Press (2016). p. 59–126 doi: 10.1016/bs.aivir.2016.08.008

3. Chen Y-N, Loa CC, Ababneh MM-K, Wu CC, Lin TL. Genotyping of turkey coronavirus field isolates from various geographic locations in the unites states based on the spike gene. *Arch Virol* (2015) 160:2719–2726. doi: 10.1007/s00705-015-2556-2

4. Valastro V, Holmes EC, Britton P, Fusaro A, Jackwood MW, Cattoli G, Monne I. S1 gene-based phylogeny of infectious bronchitis virus: an attempt to harmonize virus classification. *Infect Genet Evol* (2016) 39:349–364. doi: 10.1016/j.meegid.2016.02.015
